# Telehomecare Monitoring for Patients Receiving Anticancer Oral Therapy: Protocol for a Mixed Methods Evaluability Study

**DOI:** 10.2196/63099

**Published:** 2025-01-20

**Authors:** Dominique Tremblay, Thomas Joly-Mischlich, Annick Dufour, Marie-Claude Battista, Djamal Berbiche, José Côté, Marco Décelles, Catherine Forget, Brigitte Guérin, Manon Larivière, Frédéric Lemay, Manon Lemonde, Éric Maillet, Nathalie Moreau, Michel Pavic, Sara Soldera, Catherine Wilhelmy

**Affiliations:** 1 Nursing School Faculty of Medicine and Health Sciences Université de Sherbrooke Sherbrooke, QC Canada; 2 Department of Pharmacy Centre Intégré Universitaire de Santé et Services Sociaux de l'Estrie-Centre Hospitalier Universitaire de Sherbrooke Sherbrooke, QC Canada; 3 Department of Pharmacy Centre Intégré de Santé et Services Sociaux de la Montérégie-Centre Greenfield Park, QC Canada; 4 Faculty of Medicine and Health Sciences Université de Sherbrooke Sherbrooke, QC Canada; 5 Faculty of Nursing Université de Montréal Montréal, QC Canada; 6 Quebec Cancer Foundation Montréal, QC Canada; 7 Department of Specialty, Surgical and Cancer Services Centre Intégré Universitaire de Santé et Services Sociaux de l'Estrie-Centre Hospitalier Universitaire de Sherbrooke Sherbrooke, QC Canada; 8 Faculty of Health Sciences Ontario Tech University Oshawa, ON Canada; 9 Department of Oncology Centre Intégré de Santé et Services Sociaux de la Montérégie-Centre Greenfield Park, QC Canada; 10 Department of Hemato-Oncology Centre Intégré Universitaire de Santé et Services Sociaux de l'Estrie-Centre Hospitalier Universitaire de Sherbrooke Sherbrooke, QC Canada; 11 Cedars Cancer Centre McGill University Health Centre Montréal, QC Canada; 12 Centre de Recherche du Centre Hospitalier Universitaire de Sherbrooke Sherbrooke, QC Canada

**Keywords:** telehealth, virtual care, telehomecare monitoring, anticancer oral therapy, oncology, electronic patient-reported outcomes, electronic patient-reported experience, evaluability study, mixed methods, implementation.

## Abstract

**Background:**

Telehomecare monitoring (TM) in patients with cancer is a complex intervention. Research shows variations in the benefits and challenges TM brings to equitable access to care, the therapeutic relationship, self-management, and practice transformation. Further investigation into these variations factors will improve implementation processes and produce effective outcomes.

**Objective:**

This study aims to concurrently analyze implementation and evaluate the effectiveness of TM for patients receiving anticancer oral therapy. The objectives are to (1) contextualize how and why TM is implemented according to (a) site characteristics, (b) team characteristics, and (c) characteristics of patients receiving anticancer oral therapy; (2) assess TM effectiveness for recording electronic patient-reported outcome measures (ePROMs) and patient-reported experience measures (ePREMs) according to the site, implementation process, and patient characteristics; (3) describe the acceptability and feasibility of TM from the perspectives of the people directly or indirectly involved and provide evidence-based actionable guidance in anticipation of provincewide implementation.

**Methods:**

This type II hybrid effectiveness-implementation study uses a concurrent mixed methods design. Evaluability assessment is integrated into an emerging practice in 3 participating sites to enable the evaluation of implementation strategies on TM clinical outcomes. Quantitative data for ePROMs and ePREMs will be collected using validated oncology questionnaire. Descriptive statistics and repeated measures using multiple linear mixed models and generalized estimating equations analyses will be undertaken alongside interpretive descriptive coding of qualitative data. Qualitative data will be gathered from key informants guided by the RE-AIM (reach, efficacy, adoption, implementation, maintenance) framework and its extension, PRISM (practical robust implementation and sustainability model). The concurrent approach allows results at multiple stages of this study to be integrated iteratively. The methodological choice aims to provide real-world data that are rigorous, rapidly usable in practice, and transferable to other settings.

**Results:**

Questionnaires were pretested and the technological platform was codeveloped with members of the cancer care team and patients. Preparatory work was carried out to configure the TM platform and activate coordinating mechanisms between members of the cancer care team, patients, information technology experts, and the research team. A steering committee with 3 working groups was established to oversee the technological, clinical, and evaluation aspects of this study. Recruitment of patients for ePROMs started in February 2024, and data collection is expected to continue until March 2025. Interviews with members of the cancer care team began in November 2024. Full analysis should be completed by September 2025.

**Conclusions:**

This study will clarify how, why, for whom, and under what conditions TM can complement current care models. Our evaluability assessment will help to address implementation complexities and better understand intervention-to-practice operationalization so that implementation might be adapted to contextual factors without potentially harmful or inequitable impacts on patients.

**International Registered Report Identifier (IRRID):**

DERR1-10.2196/63099

## Introduction

### Background

Use of anticancer oral therapy, administered alone or in combination, is increasing rapidly in patients with cancer [1,2]. Anticancer oral therapy offers patients the convenience of taking medication at home, which has considerable benefits (eg, reduced hospital visits, time spent in waiting rooms and transport, transportation costs, as well as increased autonomy) [3,4]. However, there are important concerns around nonadherence to treatment [5] and the transfer of responsibility for drug administration, management of side effects, and monitoring of warning signs from the members of the cancer care team to the patients and primary care professionals, who are not always comfortable with these roles. Interdisciplinary cancer care teams (oncologists, nurses, pharmacists, psychologists, social workers, and others) are part of multiteam systems [6] that include primary care professionals (family physicians, nurses, social workers, and community pharmacists), patients, lay caregivers, nonprofit community organization workers, managers, and policy makers. Not everyone is necessarily prepared for the role and practice changes required by telehomecare monitoring (TM) [4,7,8]. Several conditions must be met to assure the safety of patients receiving anticancer oral therapy. For example, patients require capacities for self-management and adherence to treatment, the support of a family member or lay caregiver, and timely access to members of the cancer care team when needed. These team members need to establish regular and proactive monitoring, as well as effective communication and coordination, to ensure prompt response in case of need [3,7,9]. The proposed study aligns with priorities for research on TM [10] by looking specifically at its use among patients receiving anticancer oral therapy. This study focuses on the importance of structuring the increasing use of TM in such a way as to maintain the therapeutic relationship and optimal communication between patients and members of the cancer care team. The proposed structure involves a digital platform that is easy for patients to use and is regularly monitored by members of the cancer care team and alerts them when follow-up is required. This study addresses the integration into clinical processes and the effectiveness of TM on patient-reported outcomes and experience of receiving anticancer oral therapy treatments [11] based on differences in setting, team, and patient characteristics.

There are multiple terms (eg, telecare, virtual care, and eHealth) and definitions of telehealth that frequently overlap [12]. In this study, the term TM is used to describe interactions between patients in their own homes and members of the cancer care team. Telehealth generally refers to a health or social service provided at a distance using synchronous or asynchronous videoconferencing, digital applications, or telephone to improve health [11,13]. Telehealth is not new, but before the COVID-19 pandemic, its use was uncommon in cancer care and limited in most health systems [14]. For example, a 2019 study reported that only 0.6% (3/464) of family physicians in Québec, a province in Canada, had ever used telehealth, compared to 4.2% (108/2569) of the respondents in the rest of Canada [15]. Little data are available on the postpandemic use of telehealth in Canada. A national survey of 1038 participants in the United States found that the proportion of professionals in cancer care teams (oncologists, nurses, and residents) using telehealth increased from 19% to 84% during the COVID-19 pandemic [16].

In the oncology context, a systematic review (2017-2020; where, it should be noted, only 3 of 37 studies were conducted in Canada) concluded that telehealth is appropriate for symptom evaluation in adult patients during active treatment and survivorship [17]. Studies report several telehealth benefits for patients [17-20], namely improved accessibility, satisfaction, effectiveness, and comfort (especially in people with moderate symptoms or physical limitations); better adherence and treatment persistence; reductions in transportation costs and absences from work for medical follow-up; less decline in quality of life; and fewer emergency department visits. Benefits for members of cancer care teams include the potential to work remotely and thus contribute to maintaining service accessibility [21], real-time monitoring of patients-reported outcomes, and maintenance of communication among the teams involved along the care trajectory [19]. At the health system level, one study finds that telehealth can reduce missed appointments and has the potential to positively impact the efficiency of human resources [22].

However, the limitations of telehealth must also be recognized. Recent studies find that some patients prefer in-person consultations and do not wish to continue with TM after the pandemic [17], while others prefer a hybrid approach that considers their needs and preferences [21]. Some studies [20,23] warn against a model that replaces in-person visits with telehealth to reduce health care costs or deal with shortages of human resources. Other limitations include poor internet access, the cost of computers and tablets, and difficulties with remote communication, especially for people with low technological or health literacy [24]. It is crucial that recourse to telehealth does not create disparities in access to care, notably for people who may have problems using technology [17,25] and people in situations of vulnerability who require in-person psychosocial support [26]; the risk of disparities is heightened when telehealth is hastily planned, as was the case during the pandemic [24]. For members of the cancer care team, the use of TM depends on new practices, supported by legitimate leaders and dedicated resources, and a context that includes a history of successful transformation [27]. Structures must be in place to identify implementation barriers and find means of overcoming them to prevent undesirable organizational effects (eg, interpersonal conflict, lack of organizational support, burnout, intention to quit, and absenteeism) [28,29] or jeopardize the quality of care (eg, depersonalization of care, diminished partnership in care, and threats to the therapeutic relationship and relational continuity) [30]. The compatibility between TM and established practices, and perception of its usefulness, have a strong impact on TM use and on team members’ satisfaction [31]. At an institutional level, major changes require attention to organizational, legal, and ethical challenges (patient records, protection of personal information, professional responsibility, and technological evolution) [32]. To summarize, the scientific literature identifies the benefits and limitations of telehealth in cancer care. However, the diversity of participants and clinical conditions in available studies, the various research methods used, and the plurality of contexts make it difficult to grasp the nature and extent of challenges around TM implementation and effectiveness in a given context [20]. Our study aims to improve understanding of these challenges by producing evidence specific to the local context on predictors of real-world adherence to TM and its potential effects.

TM can be conceptualized as a multifaceted telehealth intervention that involves a network of heterogeneous groups (eg, patients, managers, members of the cancer care team, primary care professionals, decision makers, nonprofit community organization workers, information technology experts, and professional orders or associations), with different and even competing goals and interests and acting at different system levels. The novelty of TM differs between users. Our previous work shows that multifaceted interventions produce anticipated results when the network of people involved rally around a common objective [27], with shared leadership [33], and in partnership with patients [30].

### Theoretical and Operational Model

**Figure 1:**
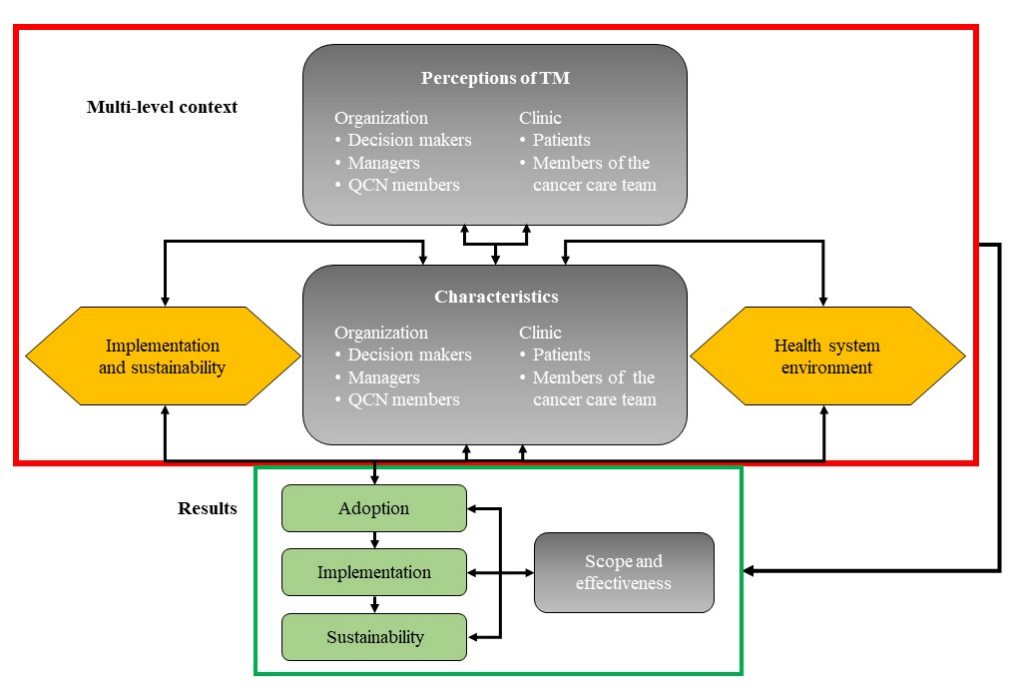
TM study framework adapted from RE-AIM/PRISM. QCN: Québec Cancer Network; PRISM: practical robust implementation and sustainability model; RE-AIM: reach, efficacy, adoption, implementation, maintenance; TM: telehomecare monitoring.

From the perspective of actor-network theory (ANT) [34,35], translating TM into practice occurs through nonlinear processes: common definition of the problem and rationale for TM (problematization), expectations and strategies to engage team and patients (interest generation), role attribution and engagement of the people involved (enrollment), coordination of network action (mobilization), and sustainability (irreversibility). These building blocks, also considered as management tools [36], are consistent with an evaluability assessment [37]. They guide our participatory approach to providing evidence-based and actionable guidance on the use of TM that will benefit patients, their caregivers, and members of the cancer care team. At the operational level, this study is based on the RE-AIM (reach, efficacy, adoption, implementation, and maintenance) framework [38], and its PRISM (practical robust implementation and sustainability model) extension [39]. The RE-AIM/PRISM framework encourages consideration of the characteristics of patients, members of the cancer care team, and their practice setting (Figure 1).

### Preparatory Work

The national telehomecare platform is supported by the Québec Ministry of Health and Social Services. The telehealth coordination office, housed in one integrated health and social services center [13], has a mandate to manage the platform’s deployment in several hospital centers. TM is based on evidence and adaptation achieved through experimentation in clinical settings. The platform was co-developed with the formal participation of stakeholders (eg, patients, managers, information technology expert collaborators, clinical leaders, and researchers). Preliminary work allowed the TM platform to be configured for use with patients [40]. Locally, its configuration was conceived and undertaken collectively by pharmacists, pivot nurses in oncology, and patients. Various strategies were implemented to support TM use to report symptoms and support self-management (eg, demonstration video, support resources, educational modules, symptom management messages, warning signs or “red flags” with decision supports, and accompaniment by survivors of cancer). Preimplementation indicators were the frequency of evaluations by patients, warning sign thresholds for clinical tools, specific alert management algorithms to guide cancer care team members’ interventions, range of monitoring (percentage of potential patients, percentage who accepted, percentage who refused, and percentage of red flags addressed), and teaching and training needs. This preliminary work was financially supported (salaries, equipment, and expertise) and closely accompanied by members of the cancer program of the Québec Ministry of Health and Social Services. The preparatory work revealed challenges in implementation (eg, identifying members of the cancer care team to assign to TM, redistributing work, leadership style, and pace of patient recruitment). Facilitating and impeding factors need to be systematically assessed to achieve expected effectiveness and guide scale-up across the province.

### Aim and Objectives

This study aims to concurrently examine the implementation process and evaluate the effectiveness of TM for patients receiving anticancer oral therapy. In line with the conceptual framework, the specific objectives are as follows:

1. To contextualize how and why TM is implemented according to (a) site characteristics (location, mandate, resources, and supports), (b) team characteristics (members of the cancer care team, managers, and coordination with primary care and nonprofit community organizations), and (c) characteristics of the patients receiving anticancer oral therapy.
2. To assess TM effectiveness for digitally recording electronic patient-reported outcome measures (ePROMs) and electronic patient-reported experience measures (ePREMs) according to the site, implementation differences, and patient characteristics.
3. To explore the acceptability and feasibility of TM from the perspective of the people directly or indirectly involved and provide evidence-based actionable guidance in anticipation of provincewide implementation.

## Methods

### Study Design

This mixed methods evaluability study uses a type II hybrid effectiveness-implementation design. An evaluability assessment is appropriate for all stages of a novel intervention [37,41,42]. It enables (1) formative assessment of clinical, organizational, and technological components through reflection “on and in practice” and rapid feedback on findings; (2) better planning; (3) accelerated integration of research findings through constant interaction between researchers and stakeholders; and (4) identification of new ways of doing things to achieve scale-up objectives [37,41,42]. A type II hybrid effectiveness-implementation design enables in-depth analysis of TM integration in a natural setting (objective 1) and effectiveness for patients receiving anticancer oral therapy (objective 2) [43,44]. Type II refers to the concurrent study of implementation and effectiveness, an approach that is recognized in cancer care interventions [45] and is consistent with the RE-AIM/PRISM framework [43] (objective 3). A mixed concurrent approach, suited to participatory evaluability assessment [37], integrates quantitative and qualitative data [46]. The voluntary participation methodology allows for the inclusion of multiple perspectives, and equity, diversity, and inclusion considerations [47]. Our study adheres to ethical research standards [48] and responsible research conduct [49]. Researchers will endeavor to maintain relationships based on trust and mutual respect by being attentive to issues around the social acceptability of health research, by protecting the identity of participants, and by involving patient partners from the very start of our study [50].

Our study comprises the following interdependent phases:

1. Preparing the ground (objective 1; duration 4 months) includes presentation of the TM study to the actors involved, development of reciprocal engagement between the research team and TM leaders including patient partners and community actors, and an environmental scan [51] to understand local dynamics (potential participants; rate of inclusion in TM; technical, professional, and personal challenges; and workforce or resource challenges). This process of collecting, interpreting, and using information from the internal and external TM environment informs strategic decision-making, guides accompaniment efforts, and ensures the progress of this study by resolving difficulties that may arise.
2. Analysis of implementation and clinical effectiveness based on RE-AIM/PRISM (objectives 1 and 2; duration 12 months) assesses achievement of TM goals and evaluates expected benefits and absence of unexpected negative effects related to its use, ePROMs and ePREMs, and resource use. Markers of TM use will be defined according to patients’ perspectives, cancer care team reorganization of work and collaboration with primary care and nonprofit community organization partners, use of human and financial resources in local arrangements, and levers and means used to overcome obstacles to safe implementation while upholding equitable access to care for patients.
3. Integrated planning for sustainability and spread (objective 3; duration 4 months) extracts lessons for eventual changes to be brought to TM to improve sustainability and future scale-up to other groups of patients (eg, intravenous therapy, immunotherapy, postsurgical cancer follow-up, palliative non–end-of-life care, and survivorship follow-up) [4]. This stage will identify the clinical, organizational, and equity challenges of TM as a complex highly context-dependent intervention. The transferability of this real-world study must also be optimized [43], especially where geographic and local dynamics differ.
4. Dissemination of results (duration 4 months) relates to the plan described in the later section of the protocol on integrated knowledge translation, for presenting findings to various groups of end users.

### Study Sites

This study will be conducted in 3 cancer centers in the province of Québec, Canada, that have introduced TM over various periods (1 year or 1 month) and dedicated varying levels of human and financial resources. These cancer centers are located in hospitals that are part of the Québec Cancer Network. These centers have distinguishing characteristics (Table 1): geographic location (mega-urban, semiurban, or urban) [52], population served, mandate (academic or community), size of the cancer care team, team caseload (number of visits or number of treatments), primary care centers (number of community service centers or number of family medicine groups), TM work redistribution between members of the cancer care team (designated leadership or shared leadership), and formal partnership with survivors of cancer trained for TM support and research.

Differences between study sites will help understand perceptions of TM in real-world contexts and the influence of various factors on adoption, implementation, effectiveness, and potential sustainability, as illustrated in Figure 1.

**Table 1:** Characteristics of participating cancer centers^a^.

		Site 1	Site 2	Site 3
**Organizational**				
	Geographic location	Mega-urban	Urban	Semiurban
	Population, n	229,359	172,713	199,376
	Mandate	Community	Academic	Community
	Size of the cancer care team	Large	Large	Small
**Clinical**				
	Radiotherapy	Yes	Yes	Yes
	Hemato-oncology	Yes	Yes	Yes
	Visits to hemato-oncology services, n	63,405	44,245	13,180
	Treatments, n	14,758	23,534	5403
**Primary care centers**				
	Community service centers, n	2	5	3
	Family medicine groups, n	8	10	8
**Implementation**				
	Patient partners, n	0	1	0
	Telehomecare monitoring coordination model^b^	Shared leadership	Designated leadership	Shared leadership

^a^Publicly accessible data (2021-2022).

^b^Coordination model apparent in early implementation.

### Participants and Sampling

This study involves key informants knowledgeable of TM. The sampling strategy for patients is nonprobabilistic, based on eligibility criteria preestablished by the cancer care team. Inclusion criteria are receiving anticancer oral therapy, being interested in TM for a minimum duration of 6 months, having internet access and experience using it, being able to report symptoms with existing tools, being open to educational material, and being able to recognize warning signs. For key informants (patients, members of the cancer care team, managers, institutional decision makers, and nonprofit community organization workers), purposive sampling [53] aims to obtain a cross-section of views from people at different decision-making levels around TM (clinical, organizational, or system). These strategies are appropriate given the exploratory nature of an evaluability study.

We estimate from hospital administrative records that between 100 and 200 patients receive anticancer oral therapy each year (N=400 in 3 participating centers). Preparatory work since March 2022 shows that about 160 of these patients would meet our inclusion criteria and about half would continue completion of the ePROMs questionnaires over 6 months (80 patients × 26 weeks × 3 times a week=6240 results) and the ePREMs experience of care questionnaire at 3 months (ePREMs: n=80). A sample of 40 participants is considered sufficient for an exploratory study of a health services intervention [54]. We estimate that about 12 individual interviews per site with patients, members of the cancer care team, and managers (n=36) would allow for theoretical saturation of qualitative data [55]. This will be supplemented by interviews with decision makers (eg, policy level, professional orders, and national organizations; n=9), for a total of 45 interviews of about 60 minutes. Participants will be offered CAD $60 (US $41.90) compensation.

### Quantitative Data Collection

Clinical data collection tools for ePROMs are based on those used in the Patient Reported Outcomes to Enhance Cancer Treatment study [56]. Ten questions, with French versions available for Canada [57], are taken from the PRO-CTCAE (Patient-Reported Outcomes Version of the Common Terminology Criteria for Adverse Events) library of the National Cancer Institute (eg, nausea or vomiting, diarrhea, constipation, dyspnea, stomatitis, skin problems, acne, hand-foot syndrome, edema, pyrexia, and more). The items are reported on a 5-point scale. Six questions on symptoms from the Edmonton Symptom Assessment System-revised are included (pain, depression, anxiety, insomnia, and fatigue) [58]. Subquestions on self-management support were created by the interdisciplinary team at the integrated health and social services center overseeing implementation to direct patients to educational modules on the TM platform. The ePROMs responses are collected 1 to 3 times per week according to patients preference. In preparatory work, 90% (144/160) preferred 3 times a week. The ePREMs questionnaire includes dimensions on health system responsiveness adapted for patients [27], satisfaction [56], and health-related quality of life [59], all measured with validated tools. Along with these dependent variables, self-reported clinical data (tumor site, time since diagnosis, treatment type, and comorbidities) and sociodemographic data (sex, gender, age, education, perceived health status, and economic situation) are collected (control variables) to enable comparison with other studies [56]. The ePREMs questionnaire will be administered 3 months after the start of TM [56]. The time for completing the questionnaire is estimated at 20 minutes, and compensation of CAD $20 (US $13.97) is anticipated. We will monitor the indicators to associate contextual factors with clinical and implementation data and promptly identify unanticipated undesirable effects of TM. These are the dependent variables of this study.

### Qualitative Data Collection

Semidirected individual interviews conducted at the mid-point in this study aim to describe enabling and impeding factors for each of the dimensions of the RE-AIM/PRISM framework, which include multilevel aspects (eg, perceptions of TM and characteristics of organizational and clinical stakeholders, the context of implementation and sustainability, and the impact of the wider health system environment) and mechanisms that support adoption, implementation, and sustainability to achieve the scope and effectiveness of TM (Figure 1). For example, questions inspired by other studies [17,56] and adapted to respondent type address the following: ease of understanding and using TM, usefulness and relevance of self-reported items, quality of communication and accessibility, clinical utility, and sense of self-efficacy for patients. Specific questions for members of the cancer care team to focus on the following: effects on their work, time management, ability to adapt their practice, adjustment of follow-up algorithms, and ways that data influence (or not) their discussions with patients. All participants will be questioned about their satisfaction and proposed improvements for scale-up and their perception of added value to the quality and safety of care.

### Data Analysis Procedure

Standard descriptive statistics will enable data to be summarized (ePROMs, ePREMs, sociodemographic, symptom severity, and percentage of indicators of the scope of TM). For ePROMs, the number of questionnaires completed will be calculated for each patient, and the number of completed weekly ePROMs questionnaires will be divided by the number of expected questionnaires. Intrasite comparisons between measures will detect differences in exposure to TM and establish key periods on the patients trajectory. Relationships between the ePROMs items and patient characteristics will be described using Spearman correlations and chi-square tests. Multiple linear mixed models and generalized estimating equations for repeated measurements on the ePROMs [60] will be used to assess relationships between patient characteristics, ePROMs items, and ePREMs dimensions. Statistical significance will be *P*<.05. These analyses will identify the most important determinants of health status, relevant indicators, and appropriate moments for measurement.

Recorded and transcribed interviews will be analyzed using a descriptive interpretive approach [61] with 2 coders independently coding meaning units. A first coding cycle will focus on themes of the RE-AIM/PRISM framework, and a second interpretive cycle will identify links between context, implementation, and effects [61]. Finally, a synthesis of this qualitative process integrating quantitative results [62] will be codeveloped with volunteers from patients, people involved in care, and the research team.

The integration (qualitative data+quantitative data) will occur at several stages: (1) methodology, with linking sampling strategies, where participants for interviews are selected from among patients receiving anticancer oral therapy; (2) data collection, by adopting a simultaneous interactive approach [63] in which emerging results will iteratively lead to adjustments to future interview questions; and (3) interpretation, in partnership with spokespersons from the various groups involved (eg, professionals, patients, lay caregivers, nonprofit community organization workers, managers, and policy makers). Attention will be paid to quality criteria for qualitative [61] and mixed methods [62] studies to mitigate study limitations.

### Ethical Considerations

This project was approved by the Research Ethics Board of the Research Centre of the Montérégie-Centre Integrated Health and Social Services Centre, which serves as the review board for all sites in this multicenter project (MP-04-2024-825, initial approval: August 29, 2023; renewed approval: June 7, 2024). This study will be conducted according to the Helsinki Declaration, and informed consent will be obtained from all individual participants [48]. Study results will be presented at the group level and will ensure that no individual, whether patients or staff, will be identifiable. Participation is voluntary and participants can withdraw from this study at any time.

### Integrated Knowledge Translation

The integrated knowledge translation plan (Textbox 1) includes approaches suited to use during and at the end of the project [64]. The research team considers it important to establish bidirectional exchanges between knowledge producers and users and involves knowledge users at all stages of this study. This approach is chosen strategically for its positive impact on the adoption of new evidence-based practices, as well as for its potential contribution to managing problems that may arise during this study. ANT provides the basis for considering the heterogeneity of people involved in TM. ANT has been widely used in applied research to study the production and use of research evidence [64]. It contributes to a better understanding of why research results do not translate easily and are not integrated rapidly into settings for which the research was produced. This is consistent with the idea that people (professionals, patients, lay caregivers, nonprofit community organization workers, managers, and policy makers) as well as context (technology platform, computers, equipment used, and funding) act on the implementation and effectiveness of TM. The plan is inscribed in a project where sustainability and scale-up are anticipated right from preliminary work. It also aims to amplify the impacts of the research at provincial, national, and international levels. As a result, the activities, format, timing of, and vehicle for dissemination will be adapted to each target audience.

**Integrated knowledge translation plan of audience and activities.**

**Actors in the field**

- Early detection of barriers, facilitators, controversies, and potential solutions
- Accessibility of the research team to actors in the field
- Discussion of interim findings in each setting
- Debriefing meetings to triage adjustments during implementation
- Newsletter published every 4 months on our website, distributed to partner networks of coresearchers and collaborators
- Support for team members for cancer and local managers
- End-of-project presentation of results in each site

**Academic and research communities and trainees**

- Generation of interest among the next generation of researchers in the renewal of cancer care: graduate students, oncologists, physicians, and health care professionals
- Dissemination of this study and its results in courses offered by research team members in their universities
- Mobilization of the coauthors’ networks
- Sharing of tools developed and found useful for consolidating patients-reported outcomes and for team resilience in facing the challenges of telehealth
- Development of evaluative, interventional, and theoretical application of actor-network theory studies and conceptual studies based on the RE-AIM (reach, efficacy, adoption, implementation, maintenance) framework and its extension, PRISM (practical robust implementation and sustainability model)

**Institutional stakeholders and knowledge brokers**

- Mobilization of partner networks at multiple decision-making levels
- Presentations at collaborator and stakeholder events
- International peer-reviewed scientific conferences
- Websites to reach a wider audience
- Publication in open-access peer-reviewed journals

**General public**

- Tailored appropriate messages to share knowledge with different interest groups and the general public as part of an open science approach to broad dissemination

## Results

An interdisciplinary steering committee including various stakeholders such as researchers, managers, members of the cancer care team, decision makers, and community actors, has been implemented to oversee the TM implementation. Its members are involved in 3 working groups that report to the steering committee; these groups will support the technological, clinical, and evaluation aspects of this study and address and assist in the resolution of controversies. At the onset of the project, our discussions with patients raised awareness that “imposing substitution on everyone could jeopardize the trust that is essential to our relationship with the cancer care team and increase the stress on us and our families; we don't want that.”

An expert committee comprising the TM leader, a pharmacist, a pivot nurse, an information technology expert, and the principal investigator supports organizational readiness, ensuring that local needs for implementation are met and that the TM platform is operational at all sites. This committee developed a PowerPoint (Microsoft Corp) presentation aiming to (1) inform members of the cancer care team and managers of the state of science about TM content, structure, and patient-professional communication mechanisms; (2) describe the potential benefits and limitations of TM for patients, members of the cancer care team, and organizations; (3) present the objectives of the evaluability assessment and effectiveness-implementation study design; and (4) describe the ePROMs monitoring platform and ePREMs questionnaire. Between November 2023 and February 2024, a total of 72 members of cancer care teams from the participating settings took part in the 90-minute session and received continuing education credits. Meanwhile, the codebook for ePROMs was developed and questionnaires for ePREMs were pretested in two cycles by a team of patient partners, researchers, and knowledge users.

Access to the TM platform by the research coordinator and principal investigator required institutional authorization and engagement to ensure adherence to strict measures to protect personal data and confidentiality. This authorization process was initiated in December 2023, with permission granted in March 2024.

Between February and July 2024, a total of 35 patients were enrolled and had completed ePROMs on the TM platform 1 to 3 times a week; 6 interviews of patients had been conducted. As of November 2024, a total of 1831 ePROMs and 41 interviews had been completed. Further, data collection with team members and other people concerned with the implementation had been initiated. The collection of quantitative and qualitative data is expected to be completed by March 2025, and the full analysis is expected to be completed by September 2025.

Preliminary interpretive coding of interviews suggested that the TM platform was easy to use and that members of the cancer care team provided prompt feedback when symptoms persisted despite self-management and educational material. Patients expressed they would like access to the TM platform on small devices that was not yet available and questioned how their oncologist was informed about the ePROMs. Clarification around the roles of nurses and pharmacists was desired to avoid confusion. The most important positive impact was the implication of a patient partner who explained the platform, how to fill in information, and could be reached for further support in using the platform.

## Discussion

### Expected Findings

This study will produce new data on the deployment of TM to support ambitions to modernize services available to patients [65]. The results will help inform decisions by integrating clinical, organizational, and health system considerations. This multilevel study will contribute to efforts to satisfy the growing appetite for telehealth, as well as its acceptability and feasibility [66]. Expected impacts are (1) a clinical contribution by informing the use of patient-reported outcomes, achievement of self-management objectives, and prompt team response to needs; (2) an organizational contribution by producing information on contextualized best practices, centered on the needs and preferences of patients, that improve access to care and appropriate use of resources and serve as a complement to and not substitution for clinical contact; and (3) a social win-win type contribution by reducing inequalities of access related to geographic or social distance, while providing essential data on opportunities and risks perceived by some groups of patients.

A recent review on telehealth reports that TM is promoted as a promising future direction for the management of anxiety and depression in patients with cancer [67]. Our study will contribute to efforts to identify types of symptoms that can be managed with TM and asynchronous interventions along with others that require in-person encounters. As patients cautioned us during the preimplementation phase, TM should not be a substitute for face-to-face interventions or be used for workforce rationalization. Additionally, our integrated knowledge translation strategy will activate the dissemination of best practices that recommend establishing the patient-professional relationship before integrating telehealth into care [68]. In the context of health human resources, TM promoters should be aware of the temptation to limit the face-to-face component of care, which may be harmful to the quality of care. Although proximity can be established through in-person or online encounters [69], patients participation [30] and collaborative governance [70] appear essential to prevent the expedient use of TM.

This study may also contribute to reducing disparities arising from the uneven use of telehealth technologies. However, other organizational priorities can increase the risk of TM program failure and increase disparities in access according to the capacities of individual patients and the availability of family caregivers to accompany them in using TM.

### Strengths and Limitations

The inclusion of 3 settings chosen for their different characteristics increases the originality of this study’s contribution. Multisite studies offer a wider perspective and will add to findings from single-institution studies on teleconsultation [17]. Our evaluability assessment will provide new empirical data on models of care that integrate TM services in advancing the quintuple aim of better health, improved care experience, well-being of members of the cancer care team, and health equity throughout the cancer journey. Our focus on how complementarity is achieved between TM and traditional face-to-face encounters aligns with calls for practical evidence in most recent reviews [17,67].

Some limitations are also anticipated in this study. The methodologies have potential biases. These include selection bias arising from convenience sampling and the fairly small sample size of participants, and observation bias due to real-world data collection that may compromise the application of results to wide-scale implementation [71]. These limitations could be compounded by the use of mixed methods [46] and by linking the implementation process to effectiveness in the specific participating sites. However, the production of such evidence provides detailed information that supports knowledge users in their decision-making and helps save time and avoid TM implementation failure.

### Conclusions

TM is a complex intervention at the interface of clinical and organizational domains. Telehealth services for patients with cancer emerged as a key theme during the COVID-19 pandemic and will only become more important in the future. Our evaluability assessment will provide new empirical data on how to design TM services that prioritize a patients-centered approach aligned with patient needs, preferences, and realistic expectations. The focus on how complementarity between TM and traditional face-to-face encounters takes shape will answer the call for practical evidence to inform implementation. Our knowledge translation plan will create opportunities to share the successes that emerge and inspire other settings. Findings may also help to improve follow-up care for patients receiving other types of anticancer treatment that require close monitoring and management. In the event of implementation failure in this study, information on contributors to failure will help prevent TM promotors from falling into some of the potential traps of implementing complex interventions.

## Appendix

Multimedia Appendix 1 Peer review report from the Accélération de la recherche et des soins pour le cancer au Québec (ACCES-Onco) (Québec, Canada).

## Abbreviations

ANT: actor-network theory
ePREM: electronic patient-reported experience measure
ePROM: electronic patient-reported outcome measure
PRISM: practical robust implementation and sustainability model
PRO-CTCAE: Patient-Reported Outcomes Version of the Common Terminology Criteria for Adverse Events
RE-AIM: reach, efficacy, adoption, implementation, maintenance
TM: telehomecare monitoring

## Data Availability

The datasets generated or analyzed during this study will not be publicly available due to ethical considerations (ie, conditions of consent received from participants) but will be available from the corresponding author on reasonable request.
