# Supplementary material for: Telehomecare Monitoring for Patients Receiving Anticancer Oral Therapy: Protocol for a Mixed Methods Evaluability Study
Source: JMIR Res Protoc. 2025 Jan 20;14:e63099. doi: 10.2196/63099 (PMC11791446; doi:10.2196/63099)
Supplement: Multimedia Appendix 1 [file resprot_v14i1e63099_app1.pdf]

Accélération de la recherche et des soins pour le cancer au Québec (ACCES-Onco)

Rapport d'évaluation

- Concours 2022-2023 -

Numéro de dossier
**326483**
**Chercheur principal/Chercheuse principale**
**Dominique Tremblay**
**Titre du projet**
Suivi virtuel en milieu de vie par téléconsultation auprès des personnes touchées par le cancer recevant un traitement contre le cancer en comprimé: une étude d'évaluabilité indispensable à l'optimisation de l'accès aux soins
**Commentaires du comité**

L'utilisation de la téléconsultation a pris une place de plus en plus importante en médecine depuis la pandémie Covid-19. Toutefois, une analyse en profondeur et rigoureuse de son utilisation et des obstacles potentiels à son utilisation demeure nécessaire. Le projet est très bien écrit avec des objectifs clairs et des données préliminaire probantes. La longueur, la quantité de l'intervention et les signalements des effets secondaires ont toutefois été perçus comme contraignants.

Quelques éléments dans l'approche méthodologique ont été soulevés, notamment dans l'évaluation de l'acceptabilité du dispositif et l'absence de groupe contrôle qui ne sont pas prises en charge par la téléconsultation. Le calcul du nombre de patients, la rémunération (risque de biais d'inclusion) et l'analyse du risque de l'utilisation de la téléconsultation demeurent discutables.

Ce projet est composé d'une solide équipe avec l'expertise nécessaire pour mener à bien cette étude. La complémentarité de l'équipe est bien en place incluant plusieurs publications dans le domaine. La présence de certains spécialistes et de patients partenaires auraient bonifié la demande.

Les données préliminaires et l'expérience de l'équipe permettront une nette accélération des connaissances sur l'utilisation de la téléconsultation. Le comité a jugé la faisabilité du projet adéquat et réalisable. Toutefois, les étapes nécessaires à atteintes des objectifs étaient nombreuses.

Les retombées sont identifiables et mesurables de manière structurée. Toutefois, l’implantabilité des résultats et des indicateurs pourrait être clarifié. La diffusion des connaissances est bien établie pour chaque étape.

Le budget est réaliste et bien justifié.

Cote	78.33
Rang	4
Nombre de demandes évaluées	15
